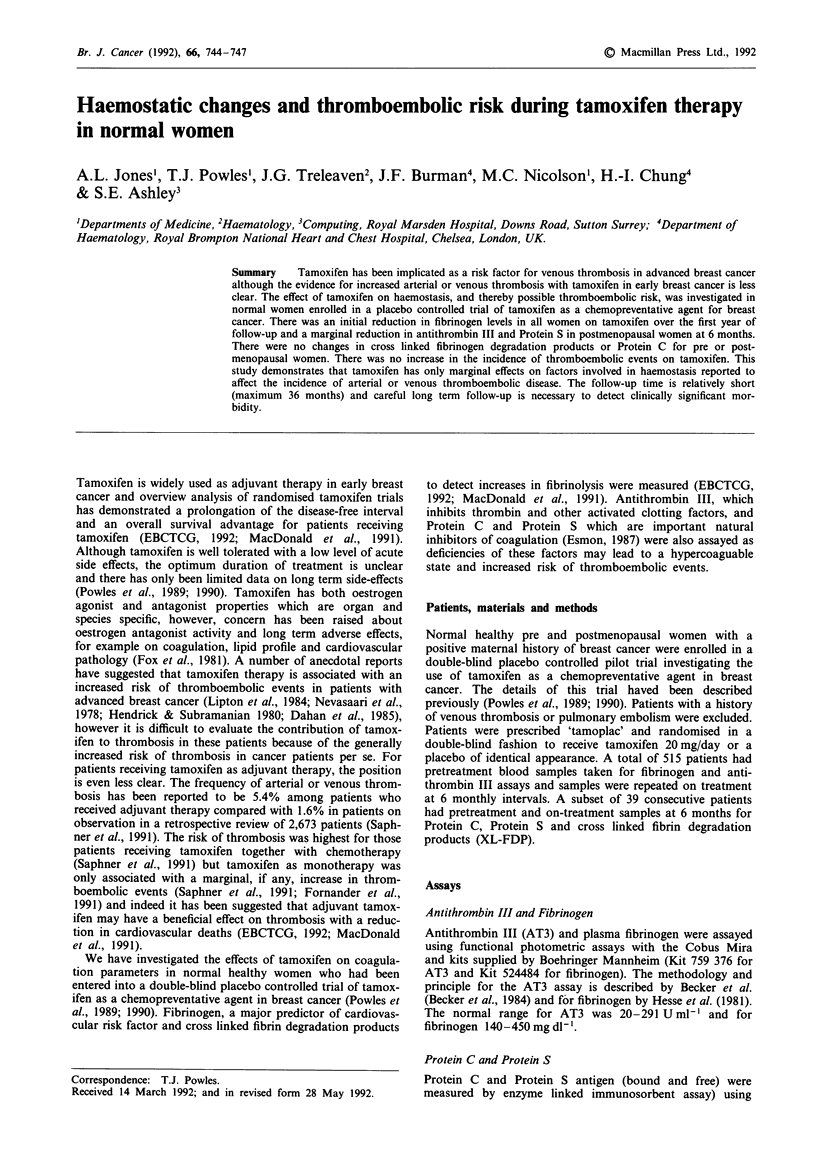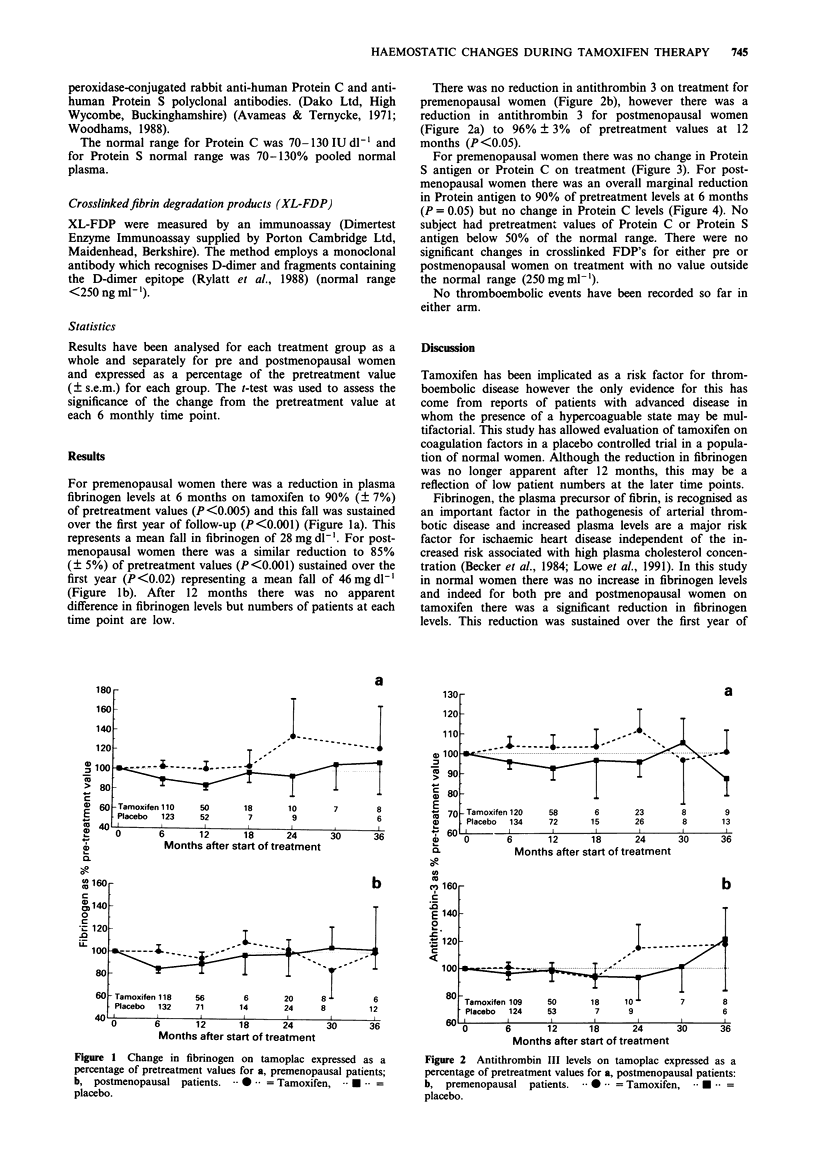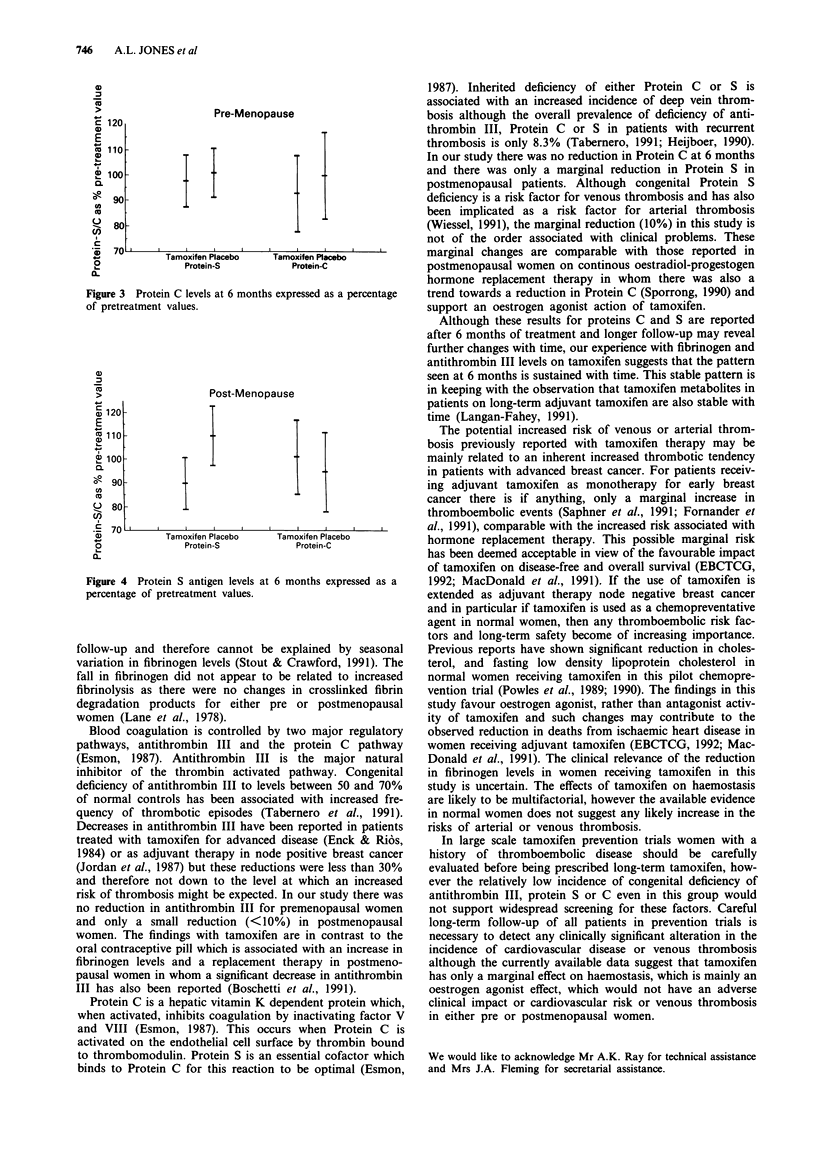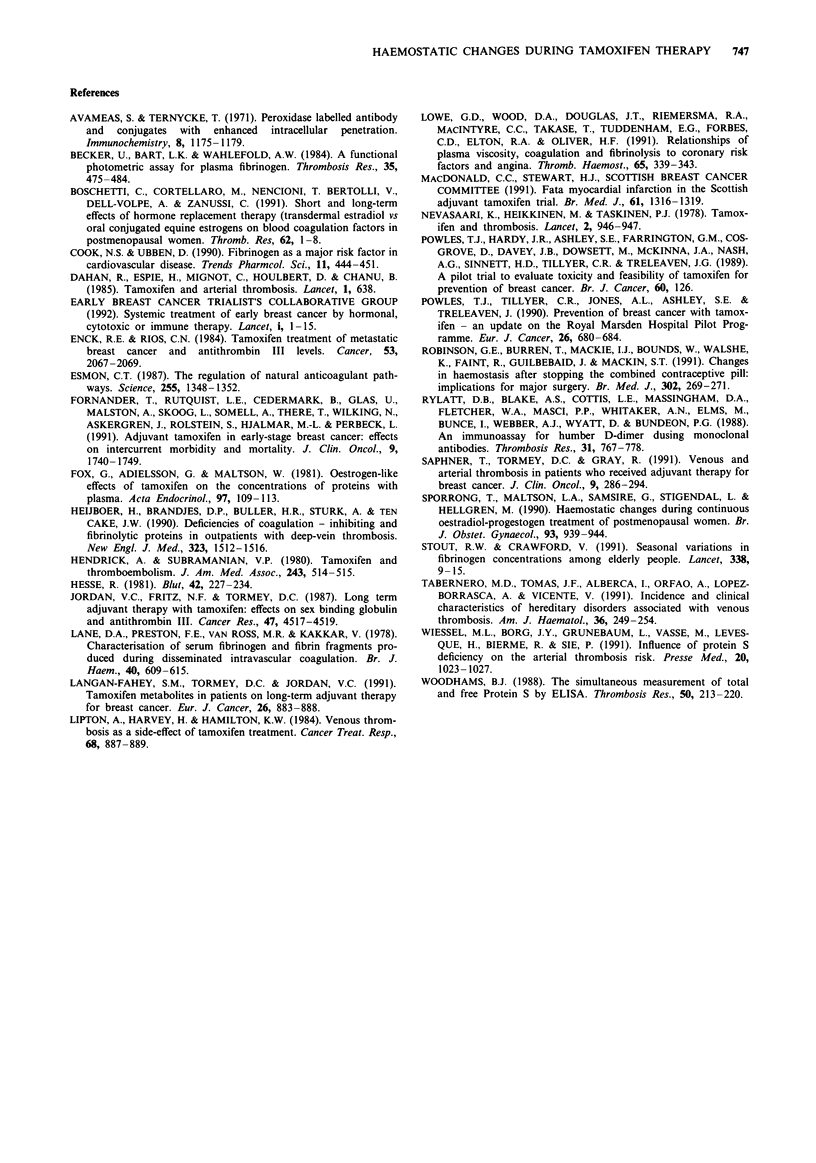# Haemostatic changes and thromboembolic risk during tamoxifen therapy in normal women.

**DOI:** 10.1038/bjc.1992.350

**Published:** 1992-10

**Authors:** A. L. Jones, T. J. Powles, J. G. Treleaven, J. F. Burman, M. C. Nicolson, H. I. Chung, S. E. Ashley

**Affiliations:** Department of Medicine, Royal Marsden Hospital, Sutton, Surrey, UK.

## Abstract

Tamoxifen has been implicated as a risk factor for venous thrombosis in advanced breast cancer although the evidence for increased arterial or venous thrombosis with tamoxifen in early breast cancer is less clear. The effect of tamoxifen on haemostasis, and thereby possible thromboembolic risk, was investigated in normal women enrolled in a placebo controlled trial of tamoxifen as a chemopreventative agent for breast cancer. There was an initial reduction in fibrinogen levels in all women on tamoxifen over the first year of follow-up and a marginal reduction in antithrombin III and Protein S in postmenopausal women at 6 months. There were no changes in cross linked fibrinogen degradation products or Protein C for pre or post-menopausal women. There was no increase in the incidence of thromboembolic events on tamoxifen. This study demonstrates that tamoxifen has only marginal effects on factors involved in haemostasis reported to affect the incidence of arterial or venous thromboembolic disease. The follow-up time is relatively short (maximum 36 months) and careful long term follow-up is necessary to detect clinically significant morbidity.


*Br. J. Cancer* (1992), **66**, 744–747 © Macmillan Press Ltd., 1992

### Haemostatic changes and thromboembolic risk during tamoxifen therapy in normal women

A.L. Jones[1], T.J. Powles[1], J.G. Treleaven[2], J.F. Burman[4], M.C. Nicolson[1], H.-I. Chung[4] & S.E. Ashley[3]

[1]*Departments of Medicine,* [2]*Haematology,* [3]*Computing, Royal Marsden Hospital, Downs Road, Sutton Surrey;* [4]*Department of Haematology, Royal Brompton National Heart and Chest Hospital, Chelsea, London, UK.*

**Summary** Tamoxifen has been implicated as a risk factor for venous thrombosis in advanced breast cancer although the evidence for increased arterial or venous thrombosis with tamoxifen in early breast cancer is less clear. The effect of tamoxifen on haemostasis, and thereby possible thromboembolic risk, was investigated in normal women enrolled in a placebo controlled trial of tamoxifen as a chemopreventative agent for breast cancer. There was an initial reduction in fibrinogen levels in all women on tamoxifen over the first year of follow-up and a marginal reduction in antithrombin III and Protein S in postmenopausal women at 6 months. There were no changes in cross linked fibrinogen degradation products or Protein C for pre or post-menopausal women. There was no increase in the incidence of thromboembolic events on tamoxifen. This study demonstrates that tamoxifen has only marginal effects on factors involved in haemostasis reported to affect the incidence of arterial or venous thromboembolic disease. The follow-up time is relatively short (maximum 36 months) and careful long term follow-up is necessary to detect clinically significant morbidity.

Tamoxifen is widely used as adjuvant therapy in early breast cancer and overview analysis of randomised tamoxifen trials has demonstrated a prolongation of the disease-free interval and an overall survival advantage for patients receiving tamoxifen (EBCTCG, 1992; MacDonald *et al.*, 1991). Although tamoxifen is well tolerated with a low level of acute side effects, the optimum duration of treatment is unclear and there has only been limited data on long term side-effects (Powles *et al.*, 1989; 1990). Tamoxifen has both oestrogen agonist and antagonist properties which are organ and species specific, however, concern has been raised about oestrogen antagonist activity and long term adverse effects, for example on coagulation, lipid profile and cardiovascular pathology (Fox *et al.*, 1981). A number of anecdotal reports have suggested that tamoxifen therapy is associated with an increased risk of thromboembolic events in patients with advanced breast cancer (Lipton *et al.*, 1984; Nevasaari *et al.*, 1978; Hendrick & Subramanian 1980; Dahan *et al.*, 1985), however it is difficult to evaluate the contribution of tamoxifen to thrombosis in these patients because of the generally increased risk of thrombosis in cancer patients per se. For patients receiving tamoxifen as adjuvant therapy, the position is even less clear. The frequency of arterial or venous thrombosis has been reported to be 5.4% among patients who received adjuvant therapy compared with 1.6% in patients on observation in a retrospective review of 2,673 patients (Saphner *et al.*, 1991). The risk of thrombosis was highest for those patients receiving tamoxifen together with chemotherapy (Saphner *et al.*, 1991) but tamoxifen as monotherapy was only associated with a marginal, if any, increase in thromboembolic events (Saphner *et al.*, 1991; Fornander *et al.*, 1991) and indeed it has been suggested that adjuvant tamoxifen may have a beneficial effect on thrombosis with a reduction in cardiovascular deaths (EBCTCG, 1992; MacDonald *et al.*, 1991).

We have investigated the effects of tamoxifen on coagulation parameters in normal healthy women who had been entered into a double-blind placebo controlled trial of tamoxifen as a chemopreventative agent in breast cancer (Powles *et al.*, 1989; 1990). Fibrinogen, a major predictor of cardiovascular risk factor and cross linked fibrin degradation products to detect increases in fibrinolysis were measured (EBCTCG, 1992; MacDonald *et al.*, 1991). Antithrombin III, which inhibits thrombin and other activated clotting factors, and Protein C and Protein S which are important natural inhibitors of coagulation (Esmon, 1987) were also assayed as deficiencies of these factors may lead to a hypercoaguable state and increased risk of thromboembolic events.

### Patients, materials and methods

Normal healthy pre and postmenopausal women with a positive maternal history of breast cancer were enrolled in a double-blind placebo controlled pilot trial investigating the use of tamoxifen as a chemopreventative agent in breast cancer. The details of this trial haved been described previously (Powles *et al.*, 1989; 1990). Patients with a history of venous thrombosis or pulmonary embolism were excluded. Patients were prescribed 'tamoplac' and randomised in a double-blind fashion to receive tamoxifen 20 mg/day or a placebo of identical appearance. A total of 515 patients had pretreatment blood samples taken for fibrinogen and antithrombin III assays and samples were repeated on treatment at 6 monthly intervals. A subset of 39 consecutive patients had pretreatment and on-treatment samples at 6 months for Protein C, Protein S and cross linked fibrin degradation products (XL-FDP).

### Assays

### *Antithrombin III and Fibrinogen*

Antithrombin III (AT3) and plasma fibrinogen were assayed using functional photometric assays with the Cobus Mira and kits supplied by Boehringer Mannheim (Kit 759 376 for AT3 and Kit 524484 for fibrinogen). The methodology and principle for the AT3 assay is described by Becker *et al.* (Becker *et al.*, 1984) and for fibrinogen by Hesse *et al.* (1981). The normal range for AT3 was 20–291 U $ml^{-1}$ and for fibrinogen 140–450 mg $dl^{-1}$.

### *Protein C and Protein S*

Protein C and Protein S antigen (bound and free) were measured by enzyme linked immunosorbent assay) using

Correspondence: T.J. Powles.
Received 14 March 1992; and in revised form 28 May 1992.

peroxidase-conjugated rabbit anti-human Protein C and anti-human Protein S polyclonal antibodies. (Dako Ltd, High Wycombe, Buckinghamshire) (Avameas & Ternycke, 1971; Woodhams, 1988).

The normal range for Protein C was 70–130 IU $dl^{-1}$ and for Protein S normal range was 70–130% pooled normal plasma.

### *Crosslinked fibrin degradation products (XL-FDP)*

XL-FDP were measured by an immunoassay (Dimertest Enzyme Immunoassay supplied by Porton Cambridge Ltd, Maidenhead, Berkshire). The method employs a monoclonal antibody which recognises D-dimer and fragments containing the D-dimer epitope (Rylatt *et al.*, 1988) (normal range <250 ng $ml^{-1}$).

### *Statistics*

Results have been analysed for each treatment group as a whole and separately for pre and postmenopausal women and expressed as a percentage of the pretreatment value (± s.e.m.) for each group. The *t*-test was used to assess the significance of the change from the pretreatment value at each 6 monthly time point.

### Results

For premenopausal women there was a reduction in plasma fibrinogen levels at 6 months on tamoxifen to 90% (± 7%) of pretreatment values ($P<0.005$) and this fall was sustained over the first year of follow-up ($P<0.001$) (Figure 1a). This represents a mean fall in fibrinogen of 28 mg $dl^{-1}$. For postmenopausal women there was a similar reduction to 85% (± 5%) of pretreatment values ($P<0.001$) sustained over the first year ($P<0.02$) representing a mean fall of 46 mg $dl^{-1}$ (Figure 1b). After 12 months there was no apparent difference in fibrinogen levels but numbers of patients at each time point are low.

There was no reduction in antithrombin 3 on treatment for premenopausal women (Figure 2b), however there was a reduction in antithrombin 3 for postmenopausal women (Figure 2a) to 96% ± 3% of pretreatment values at 12 months ($P<0.05$).

For premenopausal women there was no change in Protein S antigen or Protein C on treatment (Figure 3). For postmenopausal women there was an overall marginal reduction in Protein antigen to 90% of pretreatment levels at 6 months ($P = 0.05$) but no change in Protein C levels (Figure 4). No subject had pretreatment values of Protein C or Protein S antigen below 50% of the normal range. There were no significant changes in crosslinked FDP's for either pre or postmenopausal women on treatment with no value outside the normal range (250 mg $ml^{-1}$).

No thromboembolic events have been recorded so far in either arm.

### Discussion

Tamoxifen has been implicated as a risk factor for thromboembolic disease however the only evidence for this has come from reports of patients with advanced disease in whom the presence of a hypercoaguable state may be multifactorial. This study has allowed evaluation of tamoxifen on coagulation factors in a placebo controlled trial in a population of normal women. Although the reduction in fibrinogen was no longer apparent after 12 months, this may be a reflection of low patient numbers at the later time points.

Fibrinogen, the plasma precursor of fibrin, is recognised as an important factor in the pathogenesis of arterial thrombotic disease and increased plasma levels are a major risk factor for ischaemic heart disease independent of the increased risk associated with high plasma cholesterol concentration (Becker *et al.*, 1984; Lowe *et al.*, 1991). In this study in normal women there was no increase in fibrinogen levels and indeed for both pre and postmenopausal women on tamoxifen there was a significant reduction in fibrinogen levels. This reduction was sustained over the first year of

**Figure 1** Change in fibrinogen on tamoplac expressed as a percentage of pretreatment values for **a**, premenopausal patients; **b**, postmenopausal patients. ··●·· = Tamoxifen, ··■·· = placebo.

**Figure 2** Antithrombin III levels on tamoplac expressed as a percentage of pretreatment values for **a**, postmenopausal patients: **b**, premenopausal patients. ··●·· = Tamoxifen, ··■·· = placebo.

**Figure 3** Protein C levels at 6 months expressed as a percentage of pretreatment values.

**Figure 4** Protein S antigen levels at 6 months expressed as a percentage of pretreatment values.

follow-up and therefore cannot be explained by seasonal variation in fibrinogen levels (Stout & Crawford, 1991). The fall in fibrinogen did not appear to be related to increased fibrinolysis as there were no changes in crosslinked fibrin degradation products for either pre or postmenopausal women (Lane *et al.*, 1978).

Blood coagulation is controlled by two major regulatory pathways, antithrombin III and the protein C pathway (Esmon, 1987). Antithrombin III is the major natural inhibitor of the thrombin activated pathway. Congenital deficiency of antithrombin III to levels between 50 and 70% of normal controls has been associated with increased frequency of thrombotic episodes (Tabernero *et al.*, 1991). Decreases in antithrombin III have been reported in patients treated with tamoxifen for advanced disease (Enck & Riòs, 1984) or as adjuvant therapy in node positive breast cancer (Jordan *et al.*, 1987) but these reductions were less than 30% and therefore not down to the level at which an increased risk of thrombosis might be expected. In our study there was no reduction in antithrombin III for premenopausal women and only a small reduction (<10%) in postmenopausal women. The findings with tamoxifen are in contrast to the oral contraceptive pill which is associated with an increase in fibrinogen levels and a replacement therapy in postmenopausal women in whom a significant decrease in antithrombin III has also been reported (Boschetti *et al.*, 1991).

Protein C is a hepatic vitamin K dependent protein which, when activated, inhibits coagulation by inactivating factor V and VIII (Esmon, 1987). This occurs when Protein C is activated on the endothelial cell surface by thrombin bound to thrombomodulin. Protein S is an essential cofactor which binds to Protein C for this reaction to be optimal (Esmon, 1987). Inherited deficiency of either Protein C or S is associated with an increased incidence of deep vein thrombosis although the overall prevalence of deficiency of antithrombin III, Protein C or S in patients with recurrent thrombosis is only 8.3% (Tabernero, 1991; Heijboer, 1990). In our study there was no reduction in Protein C at 6 months and there was only a marginal reduction in Protein S in postmenopausal patients. Although congenital Protein S deficiency is a risk factor for venous thrombosis and has also been implicated as a risk factor for arterial thrombosis (Wiessel, 1991), the marginal reduction (10%) in this study is not of the order associated with clinical problems. These marginal changes are comparable with those reported in postmenopausal women on continous oestradiol-progestogen hormone replacement therapy in whom there was also a trend towards a reduction in Protein C (Sporrong, 1990) and support an oestrogen agonist action of tamoxifen.

Although these results for proteins C and S are reported after 6 months of treatment and longer follow-up may reveal further changes with time, our experience with fibrinogen and antithrombin III levels on tamoxifen suggests that the pattern seen at 6 months is sustained with time. This stable pattern is in keeping with the observation that tamoxifen metabolites in patients on long-term adjuvant tamoxifen are also stable with time (Langan-Fahey, 1991).

The potential increased risk of venous or arterial thrombosis previously reported with tamoxifen therapy may be mainly related to an inherent increased thrombotic tendency in patients with advanced breast cancer. For patients receiving adjuvant tamoxifen as monotherapy for early breast cancer there is if anything, only a marginal increase in thromboembolic events (Saphner *et al.*, 1991; Fornander *et al.*, 1991), comparable with the increased risk associated with hormone replacement therapy. This possible marginal risk has been deemed acceptable in view of the favourable impact of tamoxifen on disease-free and overall survival (EBCTCG, 1992; MacDonald *et al.*, 1991). If the use of tamoxifen is extended as adjuvant therapy node negative breast cancer and in particular if tamoxifen is used as a chemopreventative agent in normal women, then any thromboembolic risk factors and long-term safety become of increasing importance. Previous reports have shown significant reduction in cholesterol, and fasting low density lipoprotein cholesterol in normal women receiving tamoxifen in this pilot chemoprevention trial (Powles *et al.*, 1989; 1990). The findings in this study favour oestrogen agonist, rather than antagonist activity of tamoxifen and such changes may contribute to the observed reduction in deaths from ischaemic heart disease in women receiving adjuvant tamoxifen (EBCTCG, 1992; MacDonald *et al.*, 1991). The clinical relevance of the reduction in fibrinogen levels in women receiving tamoxifen in this study is uncertain. The effects of tamoxifen on haemostasis are likely to be multifactorial, however the available evidence in normal women does not suggest any likely increase in the risks of arterial or venous thrombosis.

In large scale tamoxifen prevention trials women with a history of thromboembolic disease should be carefully evaluated before being prescribed long-term tamoxifen, however the relatively low incidence of congenital deficiency of antithrombin III, protein S or C even in this group would not support widespread screening for these factors. Careful long-term follow-up of all patients in prevention trials is necessary to detect any clinically significant alteration in the incidence of cardiovascular disease or venous thrombosis although the currently available data suggest that tamoxifen has only a marginal effect on haemostasis, which is mainly an oestrogen agonist effect, which would not have an adverse clinical impact or cardiovascular risk or venous thrombosis in either pre or postmenopausal women.

We would like to acknowledge Mr A.K. Ray for technical assistance and Mrs J.A. Fleming for secretarial assistance.

### References

AVAMEAS, S. & TERNYCKE, T. (1971). Peroxidase labelled antibody and conjugates with enhanced intracellular penetration. *Immunochemistry*, **8**, 1175–1179.

BECKER, U., BART, L.K. & WAHLEFOLD, A.W. (1984). A functional photometric assay for plasma fibrinogen. *Thrombosis Res.*, **35**, 475–484.

BOSCHETTI, C., CORTELLARO, M., NENCIONI, T. BERTOLLI, V., DELL-VOLPE, A. & ZANUSSI, C. (1991). Short and long-term effects of hormone replacement therapy (transdermal estradiol *vs* oral conjugated equine estrogens on blood coagulation factors in postmenopausal women. *Thromb. Res*, **62**, 1–8.

COOK, N.S. & UBBEN, D. (1990). Fibrinogen as a major risk factor in cardiovascular disease. *Trends Pharmcol. Sci.*, **11**, 444–451.

DAHAN, R., ESPIE, H., MIGNOT, C., HOULBERT, D. & CHANU, B. (1985). Tamoxifen and arterial thrombosis. *Lancet*, **1**, 638.

EARLY BREAST CANCER TRIALIST'S COLLABORATIVE GROUP (1992). Systemic treatment of early breast cancer by hormonal, cytotoxic or immune therapy. *Lancet*, **i**, 1–15.

ENCK, R.E. & RIOS, C.N. (1984). Tamoxifen treatment of metastatic breast cancer and antithrombin III levels. *Cancer*, **53**, 2067–2069.

ESMON, C.T. (1987). The regulation of natural anticoagulant pathways. *Science*, **255**, 1348–1352.

FORNANDER, T., RUTQUIST, L.E., CEDERMARK, B., GLAS, U., MALSTON, A., SKOOG, L., SOMELL, A., THERE, T., WILKING, N., ASKERGREN, J., ROLSTEIN, S., HJALMAR, M.-L. & PERBECK, L. (1991). Adjuvant tamoxifen in early-stage breast cancer: effects on intercurrent morbidity and mortality. *J. Clin. Oncol.*, **9**, 1740–1749.

FOX, G., ADIELSSON, G. & MALTSON, W. (1981). Oestrogen-like effects of tamoxifen on the concentrations of proteins with plasma. *Acta Endocrinol.*, **97**, 109–113.

HEIJBOER, H., BRANDJES, D.P., BULLER, H.R., STURK, A. & TEN CAKE, J.W. (1990). Deficiencies of coagulation – inhibiting and fibrinolytic proteins in outpatients with deep-vein thrombosis. *New Engl. J. Med.*, **323**, 1512–1516.

HENDRICK, A. & SUBRAMANIAN, V.P. (1980). Tamoxifen and thromboembolism. *J. Am. Med. Assoc.*, **243**, 514–515.

HESSE, R. (1981). *Blut*, **42**, 227–234.

JORDAN, V.C., FRITZ, N.F. & TORMEY, D.C. (1987). Long term adjuvant therapy with tamoxifen: effects on sex binding globulin and antithrombin III. *Cancer Res.*, **47**, 4517–4519.

LANE, D.A., PRESTON, F.E., VAN ROSS, M.R. & KAKKAR, V. (1978). Characterisation of serum fibrinogen and fibrin fragments produced during disseminated intravascular coagulation. *Br. J. Haem.*, **40**, 609–615.

LANGAN-FAHEY, S.M., TORMEY, D.C. & JORDAN, V.C. (1991). Tamoxifen metabolites in patients on long-term adjuvant therapy for breast cancer. *Eur. J. Cancer*, **26**, 883–888.

LIPTON, A., HARVEY, H. & HAMILTON, K.W. (1984). Venous thrombosis as a side-effect of tamoxifen treatment. *Cancer Treat. Resp.*, **68**, 887–889.

LOWE, G.D., WOOD, D.A., DOUGLAS, J.T., RIEMERSMA, R.A., MACINTYRE, C.C., TAKASE, T., TUDDENHAM, E.G., FORBES, C.D., ELTON, R.A. & OLIVER, H.F. (1991). Relationships of plasma viscosity, coagulation and fibrinolysis to coronary risk factors and angina. *Thromb. Haemost.*, **65**, 339–343.

MACDONALD, C.C., STEWART, H.J., SCOTTISH BREAST CANCER COMMITTEE (1991). Fata myocardial infarction in the Scottish adjuvant tamoxifen trial. *Br. Med. J.*, **61**, 1316–1319.

NEVASAARI, K., HEIKKINEN, M. & TASKINEN, P.J. (1978). Tamoxifen and thrombosis. *Lancet*, **2**, 946–947.

POWLES, T.J., HARDY, J.R., ASHLEY, S.E., FARRINGTON, G.M., COSGROVE, D., DAVEY, J.B., DOWSETT, M., MCKINNA, J.A., NASH, A.G., SINNETT, H.D., TILLYER, C.R. & TRELEAVEN, J.G. (1989). A pilot trial to evaluate toxicity and feasibility of tamoxifen for prevention of breast cancer. *Br. J. Cancer*, **60**, 126.

POWLES, T.J., TILLYER, C.R., JONES, A.L., ASHLEY, S.E. & TRELEAVEN, J. (1990). Prevention of breast cancer with tamoxifen – an update on the Royal Marsden Hospital Pilot Programme. *Eur. J. Cancer*, **26**, 680–684.

ROBINSON, G.E., BURREN, T., MACKIE, I.J., BOUNDS, W., WALSHE, K., FAINT, R., GUILBEBAID, J. & MACKIN, S.T. (1991). Changes in haemostasis after stopping the combined contraceptive pill: implications for major surgery. *Br. Med. J.*, **302**, 269–271.

RYLATT, D.B., BLAKE, A.S., COTTIS, L.E., MASSINGHAM, D.A., FLETCHER, W.A., MASCI, P.P., WHITAKER, A.N., ELMS, M., BUNCE, I., WEBBER, A.J., WYATT, D. & BUNDEON, P.G. (1988). An immunoassay for humber D-dimer dusing monoclonal antibodies. *Thrombosis Res.*, **31**, 767–778.

SAPHNER, T., TORMEY, D.C. & GRAY, R. (1991). Venous and arterial thrombosis in patients who received adjuvant therapy for breast cancer. *J. Clin. Oncol.*, **9**, 286–294.

SPORRONG, T., MALTSON, L.A., SAMSIRE, G., STIGENDAL, L. & HELLGREN, M. (1990). Haemostatic changes during continuous oestradiol-progestogen treatment of postmenopausal women. *Br. J. Obstet. Gynaecol.*, **93**, 939–944.

STOUT, R.W. & CRAWFORD, V. (1991). Seasonal variations in fibrinogen concentrations among elderly people. *Lancet*, **338**, 9–15.

TABERNERO, M.D., TOMAS, J.F., ALBERCA, I., ORFAO, A., LOPEZ-BORRASCA, A. & VICENTE, V. (1991). Incidence and clinical characteristics of hereditary disorders associated with venous thrombosis. *Am. J. Haematol.*, **36**, 249–254.

WIESSEL, M.L., BORG, J.Y., GRUNEBAUM, L., VASSE, M., LEVESQUE, H., BIERME, R. & SIE, P. (1991). Influence of protein S deficiency on the arterial thrombosis risk. *Presse Med.*, **20**, 1023–1027.

WOODHAMS, B.J. (1988). The simultaneous measurement of total and free Protein S by ELISA. *Thrombosis Res.*, **50**, 213–220.